# TUMIR: an experimentally supported database of microRNA deregulation in various cancers

**DOI:** 10.1186/2043-9113-3-7

**Published:** 2013-04-17

**Authors:** Lei Dong, Min Luo, Fang Wang, Junwu Zhang, Tingting Li, Jia Yu

**Affiliations:** 1 Department of Biochemistry, Institute of Basic Medical Sciences, Chinese Academy of Medical Sciences (CAMS) & Peking Union Medical College (PUMC), National Laboratory of Medical Molecular Biology, Beijing, 100005, PR China; 2 Department of Biomedical Informatics, Peking University Health Science Center, Beijing, 100191, China

**Keywords:** MicroRNA, Cancer, Database

## Abstract

**Background:**

MicroRNAs were found to play an important role in cancers and several literatures exist to describe the relationship between microRNA and cancer, but the expression pattern was still faintly. There is a need for a comprehensive collection and summary of the interactions under experimental support.

**Description:**

TUMIR (http://www.ncrnalab.com/TUMIR/), a manually extracted database of experimentally supported microRNA-cancer relationship, aims at providing a large, high-quality, validated comprehensive resource of microRNA deregulation in various cancers. The current version includes a systematic literature search to May-1-2012 using PubMed database, contains data extracted from 205 literatures and 1163 entries describing a regulatory interaction between human microRNAs and cancers. Each entry in the database contains the details of microRNA name, the disease name, case number, control number, p value, the experimentally validated targets, sample type, and a brief description of patients’ clinic pathologic parameters mentioned in the same paper. The website has several extensive external links to the related websites and any requests can be made by emailing to tumir_pumc@163.com.

**Conclusion:**

TUMIR is an open access website and will be an accurate clue for the researchers who are interested in better understanding the relationship between miRNAs and cancer.

## Background

MicroRNAs (miRNAs) are a class of approximately 22–25 nt long endogenous single-stranded RNAs that generally repress the expression of mRNAs. They control mRNA degradation and/or translation through employing a silencing complex to target the 3' UTR region of the mRNAs [1]. In the past decades, miRNAs were found to play an important role in a wide range of biological processes and human diseases especially in tumors [2-4].

Since hundreds of miRNAs were predicted and identified in human cells and tissues, a lot of miRNA-related databases have been built. Of them, several resources provide complete annotation and nomenclature such as miRBase [5], miRGator [6] and miRGen [7]. Several provide experimentally validated microRNA information such as TarBase [8], miR2Disease [9], dbDEMC [10], miRCancer [11] and miRecords [12]; And also several provide computational predictions such as TargetScan [13], PicTar [14] and microRNA.org [15]. TUMIR is mainly focusing on the data with experimental validation and all samples mentioned in this database must be collected from human. In contrast to TUMIR, the dbDEMC [10] retrieved miRNA-cancer relationship mainly from the analysis of high-throughput expression data. miR2Disease [9], the same as miRCancer [11], integrated data from various experiments without distinguishing human samples from cell lines. Meanwhile, TUMIR is also different from TarBase [8] and miRecords [12] which mainly focused on the miRNA targets. In a word, TUMIR provides a soild evidence of the miRNA deregulation in various cancers.

Accumulating evidence is emerging that miRNAs are closely involved in human cancer initiation, progression, prognosis and diagnosis. Moreover, some miRNAs have been shown to dysregulate and function as tumor suppressors or oncogenes in many kinds of cancers. For example, miR-21 is frequently upregulated in human hepatocellular cacinomas and lung cancer [16-18], and miR-29b is down-regulated in acute myeloid leukemia [19], and liver cancer [20]. Actually, many groups conducted the same experimental designation to analyze miRNA expression through miRNA array, real-time PCR, northern blot or other methods in human tissues or cells. However, the accuracy of miRNA expression might be interfered by different materials and methods used in these experiments, For example, miR-21, which has been recognized as an oncomir in most of cancers, did not show any differences between case and control groups in some experiments [21]; miR-375, which was found to be down-regulated in multiple cancers, was shown to be up-regulated in some cancers [22-24]. Therefore, finding a clear expression pattern in certain cancer remains a challenging task, especially a systematic documentation and analysis of such experimentally supported miRNA-cancer relationship is needed. To this aim, we have developed a manually extracted database: TUMIR, which provides a large, high-quality, and experimentally validated comprehensive resource of miRNA deregulation in various cancers.

The TUMIR database contains the relationship between miRNAs and cancer, together with the parameters of the design of experiments and patients’ clinic pathologies. Additionally, the website has several extensive links, such as a direct link to the paper’s information from the NCBI [25] website or a hyper link to the miRNA’s information from miRBase [5].

## Construction and content

### Data sources

We conducted a systematic literature search to May-1-2012 using PubMed database, by means of the terms: “cancer”, “carcinoma” and “neoplasm” in combination with the keywords “microRNA” and “miRNA”. All results were limited to studies of human. We also searched the reference lists of eligible studies and relevant reviews. Reports fulfilling the following criteria were included if: (i) they were miRNA profiling studies in patients with cancer; (ii) they used cancer and noncancerous samples for comparison; (iii) they did not only use high-throughput methods; (iv) there are more than three samples in the studies and when they got the expression values, the samples could not be pooled. Thus, miRNA profiling studies using only cell lines, or only focusing on the utilization of microarrays, sequencing methods were not included.

## Implementation

All the data were stored in a series of relational database implemented with MySQL 5.5.0 on windows system. The front-end web interface implementation is based on PHP and Javascript running under Apache 2.2.21. APIs were generated by java version 1.7.0_15.

The current release of TUMIR contains data extracted from 205 literatures and 1163 entries describing regulatory interactions between human miRNAs and cancer, and complete citations are also provided. The TUMIR data set contains the expression pattern of miRNAs that were up-regulated, down-regulated or no significant changes in various cancers. Each entry in the database contains the details of miRNA name, the disease name, case number, control number, p value, the experimentally validated targets, sample type (tissue, serum, etc.) and a brief description of patients’ clinicopathologic parameters mentioned in the same paper. TUMIR provides a user-friendly interface for searching each entry by miRNA name or cancer name.

## Utility and discussion

### Search page

The TUMIR is designed to serve as a search engine to query of confirmed miRNA-cancer relationship with experimental support. It provides a drop-down list search function. Users can query the database by choosing a miRNA name or cancer type. Once a certain miRNA or cancer type is received as a key word, the search engine will search all the items that contain the query word in TUMIR database. For instance, hsa-miR-375 was reported in a lot of original publications and it was hard to make a full understanding of its expression pattern in various cancer. When users plan to query the expression pattern in all kinds of cancer, they can choose the key word “hsa-miR-375” from the “Search by miRNA Name” drop-down list and retrieve all the systematic documentation of the selected miRNA. After that, a“search results" web page emerges and shows all of information involved in “hsa-miR-375” from queried cancer papers. Each paper is introduced as an individual table, which includes multiple items representing differently experimental data. In the "search results" page, users are able to freely choose the items they are interested in by marking the checked box before the detailed information. They also can click "CHECK_ALL" button located at the top or bottom of the tables to select all of items listed in these tables, or click "CANCEL" button to quit all of selections. However, users have to select at least one item before they press "Submit" button to access the next step. In addition, clicking the "reference" button under the hyperlink of title will lead to the full reference information of the paper (author, date, magazine, etc.). In this page, clicking the "Submit" button will show the summary information of the items you chose. Different expression patterns of miRNAs in cancer are separated by colors. Users will find the relationship between this miRNA and certain cancer by clicking the hyperlinks at the top of tables directly.

**Figure 1:**
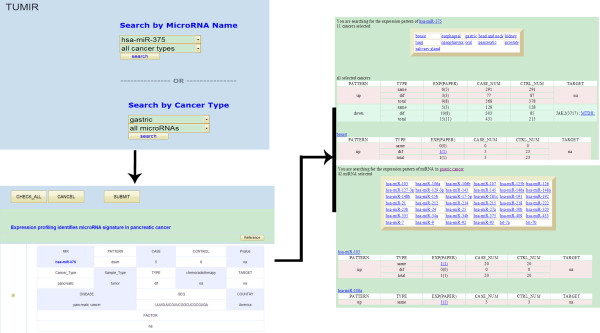
A schematic workflow of TUMIR.

Similar to search by miRNA name, users can choose certain cancer from the “Search by Cancer Type” drop-down list, such as “gastric” cancer. Users can also choose their interested items in the "search results" page and click “Submit” button to find the summary information. Different from searching by miRNA name, the data here is organized by selected cancer and different miRNAs (Figure 1).

### Statistics page

The statistics page currently demonstrates 4 histograms of the number of miRNAs and samples associated with multiple cancers archived in TUMIR. These histograms summarized the status of miRNA-cancer researches.

### Help page

We employed a user case and a user guide here to make researchers better understand the usability of our website. TUMIR also offers a submission part in this page that allows other researchers to submit un-documented miRNA-cancer patterns. Once accepted by the submission checkup team, the submitted resources will be updated into the database, and will be available to the public in the next release. TUMIR will be updated bimonthly.

### Download page

All of the data described above is freely available for download in TUMIR according to the GNU Public License. Users who want to download the current release or previous versions of TUMIR will be taken to the “download” page by clicking “Download” button from the top menu bar. These data are available in multiple formats, such as .sql, .xls and a tab-delimited format. We also provide a series of APIs here for batch search, researches can download the APIs, javadocs and a test program here.

## Conclusions

The current version of TUMIR attempted to build a widely accessible and user-friendly database that connects miRNAs with cancer through their experimentally supported expression patterns. Although the precise molecular mechanism surrounding many miRNAs and cancer requires further clarification, TUMIR can provide us accurate clue to better descript the relationship between miRNAs and cancer. In order to provide a central resource for the doctors who wish to find some diagnostic markers of certain cancer or for the researchers who wish to study the function and molecular mechanism of miRNAs in some cancer, we not only continuously expand the numbers of known and newly identified items but also create more external links to related websites. We also allow other researchers to contribute to the data contents. Recently, long non-coding RNAs (lncRNAs) in mammals were found to be powerful regulators of gene expression in normal and pathological conditions [26-29]. Similar to miRNAs, lncRNAs have been shown to play vital roles in cancer, and their expression and function are largely unknown [26]. In order to satisfy these requirements, we plan to make rapidly updates and modifications to present a new version of TUMIR.

## Availability and requirements

### Availability

The TUMIR database can be directly accessed from the web page: http://www.ncrnalab.com/TUMIR/. This database is publicly and freely accessible, requiring no registration and with no restrictions on use.

## Technical requirements

It is recommended that one of the following browsers is used: Mozilla Firefox 3 or Safari 4 on Linux, Mac OSX or Windows, Chrome on Linux or Windows, Internet Explorer 8 on Windows. In order to use the APIs, the java complier compliance level should be 1.7.

## Competing interests

The authors declare that they have no competing interests.

## Authors’ contributions

LD, ML, FW and JY performed design and analysis and wrote the manuscript. LD and ML are involved in the data integration and maintenance of the database. T-T L is responsible for bioinformatics analysis. JY, T-T L, J-W Z and LD designed the study. All authors have read and approved the final manuscript.
